# THE SYNERGY OF QUALITATIVE AND MIXED METHODS TO EXPLORE THE RELATIONSHIP BETWEEN OLDER ADULTS AND THEIR SENSE OF PLACE

**DOI:** 10.1093/geroni/igz038.2044

**Published:** 2019-11-08

**Authors:** Joyce Weil

**Affiliations:** University of Northern Colorado, Colorado, United States

## Abstract

Understanding person-environment fit, or how well older adults’ environment suits their needs, is complex. While all methodological approaches to gain deeper knowledge about fit have great value, the combination of methodologies provides a layered account of the experience of aging in a place, including the people older adults find important. This research found a clear benefit of combining results of 2 focus groups, 85 in-depth qualitative interviews, and 100 web-based surveys to assess how older adults discuss who and what is meaningful to them. Results show the combined benefit of using a qualitative and mixed-method process during the psychometric testing of the Person-Place Fit Measure for Older Adults. Qualitative focus groups and interviews offered additional domains and exact language older adults used to discuss place. Early qualitative phases improved later stages of this research, namely, online testing, reduction of survey items, and psychometric testing of the final quantitative instrument.

